# Metastatic breast carcinoma of the coracoid process: two case reports

**DOI:** 10.1186/1749-799X-5-22

**Published:** 2010-03-26

**Authors:** Eric C Benson, Darren S Drosdowech

**Affiliations:** 1 Department of Orthopaedic Surgery and Rehabilitation, Division of Shoulder and Elbow Surgery, MSC10 - 5600, 1 University of New Mexico, Albuquerque, NM 87131, USA; 2 University of Western Ontario, Division of Orthopedic Surgery, Hand and Upper Limb Centre, St. Joseph's Health Centre, 268 Grosvenor St, London, ON N6A 4V2, Canada

## Abstract

**Background:**

The coracoid process of the scapula is a rare site of involvement for metastatic disease or for primary tumors. We are unaware of any reports in the literature of pathologic coracoid process fractures and only one report of metastatic disease to the coracoid.

**Methods and Results:**

In this case report, we present two cases with metastatic breast carcinoma of the coracoid process, one of which presented with a pathologic fracture of the coracoid.

**Conclusions:**

An orthopaedic surgeon must be aware of the potential for metastatic disease to the coracoid as they may be the first medical provider to encounter evidence of malignant disease.

## Introduction

The coracoid process of the scapula is a rare site of involvement for metastatic disease or for primary tumors. Bone metastases are common in patients with breast carcinoma, with an incidence as high as 73% (range 47-85%) [1]. The exact mechanism of metastases to bone remains unknown.

We are unaware of any reports in the literature of pathologic coracoid process fractures, and only one report of metastatic disease to the coracoid [2]. We present the cases of two patients with metastatic breast carcinoma of the coracoid process, one of which presented with a pathologic fracture of the coracoid. We informed the patients or their families that the data concerning their cases would be submitted for publication, and they consented.

## Case 1

**Figure 1:**
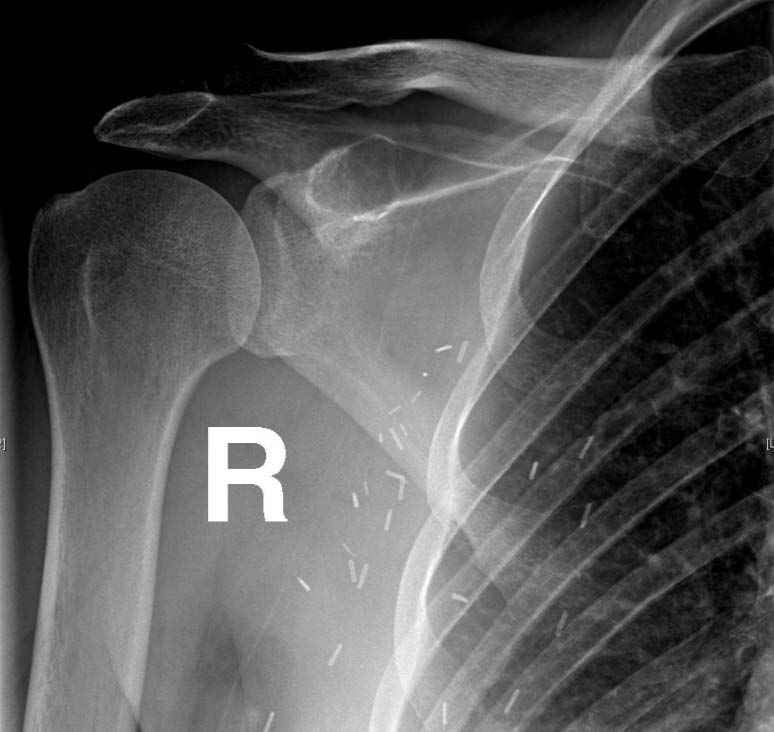
**AP radiograph demonstrating the metastatic lesion of the coracoid process**.

**Figure 2:**
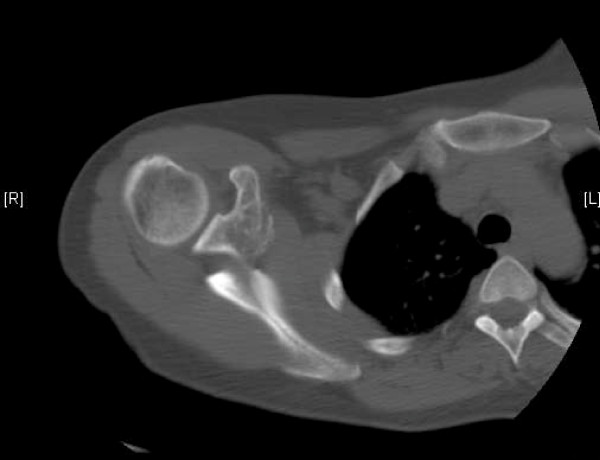
**CT scan showing the metastatic lesion at the base of the coracoid**.

A 40-year-old, right-hand dominant female who had a known history of right breast carcinoma presented to our clinic for evaluation for open biopsy of a lesion at the base of the coracoid. Four months prior to clinic presentation, she underwent right breast lumpectomy and lymph node dissection. Surgical pathology revealed invasive mammary carcinoma, SBR grade 2 with no involvement of the lymph nodes. Resection margins were negative. She was Her-2-neu negative, estrogen receptor negative, and progesterone receptor positive. A bone scan revealed increased uptake at the eighth thoracic vertebra and in the region of the coracoid in the right shoulder. Further CT imaging of both regions indicated a fracture through the transverse process of T8, though the patient was asymptomatic at this level and had a prior history of a fall from a horse that correlated with this finding. There was no history of any shoulder pain resulting from or subsequent to that fall. CT imaging of the scapula showed osteolytic change at the base of the coracoid. Radiographs and relevant CT scan images are shown (Figures 1 and 2). She had received the first cycle of adjuvant chemotherapy with FEC-100 but further cycles were discontinued until further information regarding the possible sites of metastases was collected. Instead, she was placed on Tamoxifen and Clodronate. She was otherwise healthy and took no other medications.

On physical exam there was no palpable mass in the region of the right shoulder, no skin discoloration or changes, and her range of motion and strength were normal. She was nontender to palpation over the coracoid process. She had no tenderness to palpation over T8 or elsewhere throughout the spine. Upper and lower extremity neurovascular exam showed no focal deficits.

The patient consented to open biopsy of the coracoid and was taken to the operating room. Through a deltopectoral approach, the coracoid was identified and biopsy specimens from the lesion at the base of the coracoid were sent to pathology for frozen section and permanent sections. The intra-operative frozen section was positive for adenocarcinoma.

The patient had no complications following the biopsy and the surgical pathology report confirmed the lesion was a metastatic breast adenocarcinoma. The immunohistochemical stains showed moderately to strongly positive progesterone receptors in about 15% and moderately positive estrogen receptors in about 2% of malignant cells.

Approximately twenty months after her initial lumpectomy, the patient underwent right partial mastectomy for recurrent carcinoma. At most recent follow-up, two years after initial diagnosis, she is doing well with no evidence of local recurrence or progression of metastatic disease.

## Case 2

A 23-year-old, right-hand dominant female sports coach fell backwards onto outstretched arms while snowboarding one week prior to presentation. She noted immediate left shoulder pain, was seen at on outside Emergency Department, and was referred to orthopedics for management of her shoulder injury. She sustained no other injuries in the fall. She noted no other previous complaints with regard to her left shoulder. She took Naprosyn for pain relief. Over the months leading up to the fall, she was treated with NSAIDs at another center for chest wall pain presumed to be osteochondritis. Otherwise, she had no significant findings in review of her past medical history. Prior surgeries included removal of a Bartholin's cyst.

Physical examination revealed isolated point tenderness over the tip of the coracoid. She had full neck, shoulder, and elbow range of motion with some discomfort at the terminal range of internal and external rotation of the shoulder. Her neurovascular exam showed no focal deficits.

**Figure 3:**
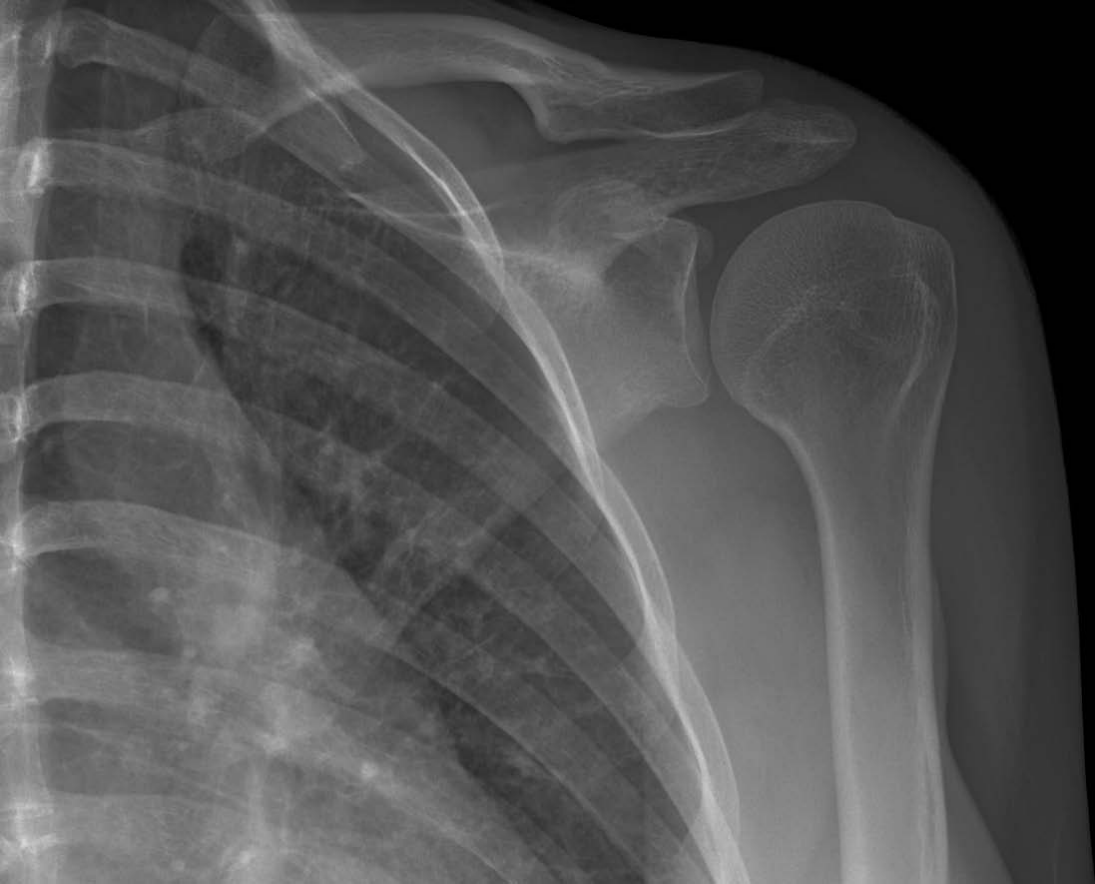
**AP radiograph of the nondisplaced pathologic coracoid process fracture**.

**Figure 4:**
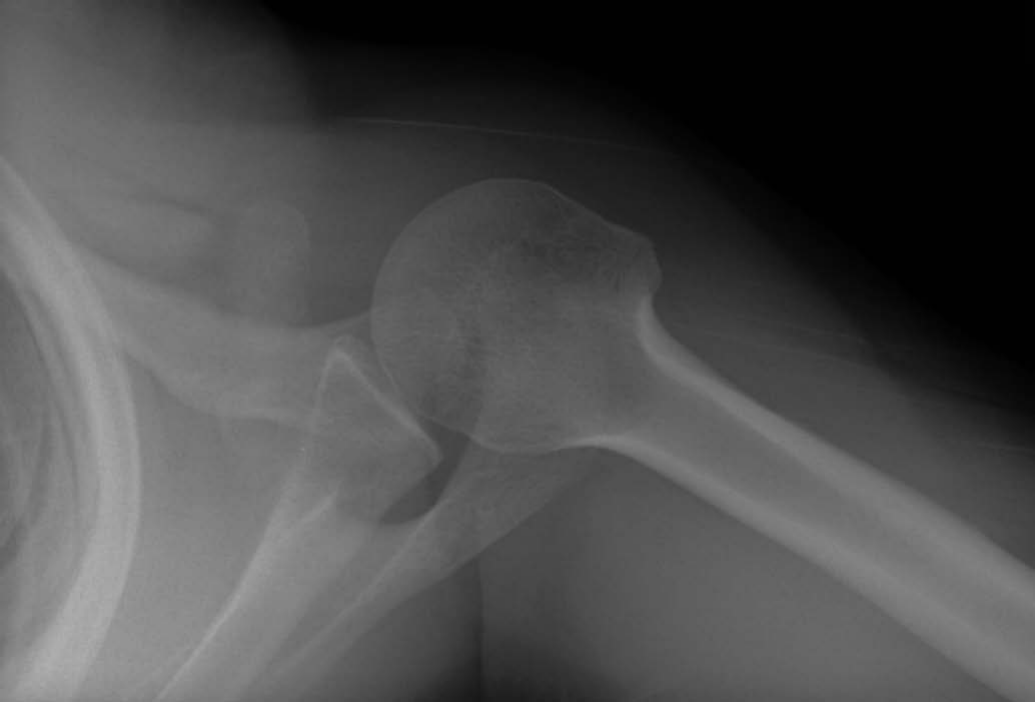
**Axillary radiograph showing the pathologic coracoid fracture**.

Radiographs showed a nondisplaced fracture of the coracoid (Figures 3 and 4). These were compared to her outside films taken immediately after her fall and showed no interval change in position of the fragment.

We recommended non-operative management of this stable injury. Short-term immobilization using a sling followed by initiation of physiotherapy was arranged. Gentle strengthening was to start after approximately four to six weeks as tolerated.

Tragically, this previously healthy, active, young woman was admitted to an outside facility only two weeks later with hypercalcemia, multiple sites of bone metastases noted on skeletal survey, and an abnormal liver scan. She was diagnosed with metastatic adenocarcinoma of the left breast. In addition to the coracoid, she had multiple metastatic lesions in her thoracic spine and bilateral femurs as well as brain and liver metastases. Over the course of the following four months she suffered from encephalopathy, SIADH, leptomeningeal carcinomatosis, and eventually passed away in her home receiving palliative care.

Though the patient's mechanism of injury was consistent with an acute coracoid fracture, in retrospect her injury was likely a pathologic fracture secondary to her metastatic breast adenocarcinoma.

## Discussion

Tumors of the coracoid process are rare. We could only identify one report of a metastatic lesion to the coracoid using a PubMed search of the literature [2]. Primary bone tumors of the coracoid include osteoid osteoma, osteosarcoma, giant cell tumor, chondrosarcoma, capillary hemangioma, aneurysmal bone cyst, lymphoma, and plasmacytoma [3]. In our PubMed literature search, we found no reports of pathologic coracoid fractures.

Breast cancer's propensity to metastasize to bone is not clearly understood. Batson described the valveless venous plexus commonly thought to contribute to the spread of breast and prostate carcinoma to sites in the axial and appendicular skeleton [4]. More recently, studies suggest some of the mechanisms for bone destruction once tumor cells have gained access to a distant site. These include osteoclast activating factors such as parathyroid hormone-related protein (PTH-rP), tumor necrosis factor (TNF) α and β, epidermal growth factor (EGF), and prostaglandins [5]. These changes to the bone architecture lead to structural weakness, and typically, the radiographic appearance of breast metastases to bone is one of mixed osteoblastic and osteolytic appearance.

Often, the orthopaedic surgeon is the first medical provider to encounter evidence of malignant disease and as such must be aware of potential sites of involvement. When interpreting radiographs, especially in an area as difficult as the coracoid, it is important to maintain an index of suspicion for underlying pathologic processes, especially since isolated fractures of the coracoid process are rare [6-23]. When present, it may be difficult to identify the bony architecture at the fracture site secondary to overlying structures. It may be prudent to obtain extra imaging to clearly show the bony characteristics of the injury. A 20 degree posterior oblique film with 20 degrees of cephalad angulation can show coracoid fractures and bone morphology more clearly if other views are inconclusive [24]. CT scans may also be useful.

The role of the orthopaedic surgeon may also include recommendations for bisphosphonate use. In concert with the consulting medical oncologist, administering bisphosphonates may reduce the risk of skeletal complications in patients receiving systemic therapy who have lytic bone metastatic lesions secondary to breast cancer [25,26].

The coracoid process of the scapula is a rare site of acute isolated trauma, primary tumors, or of metastatic disease. We present what we believe to be the first reported case of a pathologic fracture of the coracoid in one of two patients who presented with metastatic breast carcinoma of the coracoid. Although rare, orthopaedic surgeons must be aware of the potential for a pathologic process involving the coracoid.

## Consent

Informed consent was obtained from the patient or patient's family for publication of this case report and all accompanying radiographic images.

## Competing interests

The authors declare that they have no competing interests.

## Authors' contributions

DD performed all clinical evaluations and interactions with the patients. EB reviewed the case files, contacted the patients' or patients' families to obtain informed consent, and prepared the manuscript and image files. Both EB and DD read, revised, and approved the final manuscript.
